# First person – Arvind Thekkinghat

**DOI:** 10.1242/bio.047886

**Published:** 2019-09-15

## Abstract

First Person is a series of interviews with the first authors of a selection of papers published in Biology Open, helping early-career researchers promote themselves alongside their papers. Arvind Thekkinghat is first author on ‘Apolipoprotein L9 interacts with LC3/GABARAP and is a microtubule-associated protein with a widespread subcellular distribution’, published in BiO. Arvind conducted the research described in this article while a PhD student in P. N. Rangarajan's lab at the Indian Institute of Science, Bangalore, India. He is continuing his studies there, investigating eukaryotic cell biology and gene function.


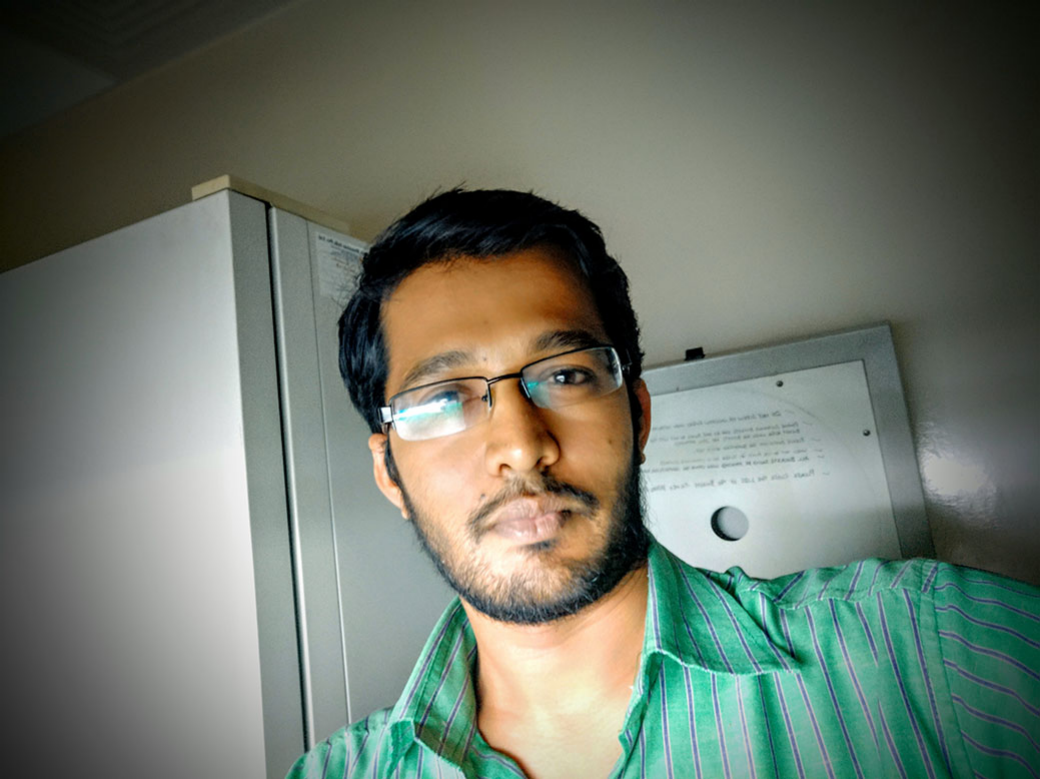


**Arvind Thekkinghat**

**What is your scientific background and the general focus of your lab?**

Both my bachelor's and master's degrees were in biotechnology, which is actually a misnomer. It is a college degree that is a mish-mash of various biology subjects like biochemistry, microbiology, cell biology and immunology, etc. It is quite popular in India and one learns a little bit of everything, giving them the opportunity to specialize later in whatever subject they like best. The general focus of our lab is eukaryotic gene expression, with special focus on yeast biology. Though I worked with a mouse protein, most of my current colleagues investigate metabolism and gene regulation in the respiratory yeast *Pichia pastoris*. My PI is quite flexible in that he generally encourages graduate students to play around with a couple of research problems before allowing them to choose whatever suits them best. I feel that's good, and it gives students a sense of independence.

**How would you explain the main findings of your paper to non-scientific family and friends?**

I often say, because it's been less than 20 years since the human and mouse genomes were fully sequenced, that it's an exciting time to work in biology and will continue to be so for a long time. A lot of genes are poorly characterized, and there's a lot of work left to be done. The protein I worked with, ApoL9, is found only in the mouse and rat genomes. I tried to answer the most basic questions, like which tissues the protein is expressed in and what compartments of the cell it might have functions in. The protein revealed its secrets to us rather slowly, and we found that it travels to a lot of compartments inside the cell. We already knew that it bound a specific type of membrane lipid. Now we know that it might regulate autophagy, which is a process by which material inside the cell is recycled. It also binds to wiry structures called microtubules that have many functions in the cell, including transport. I'm sure this is not the last we'll hear about this protein.

“It's an exciting time to work in biology and will continue to be so for a long time.”

**What are the potential implications of these results for your field of research?**

These results are likely to be of interest to researchers working on areas like lipid biology, autophagy and membrane dynamics. I don't think there are many proteins that can potentially interact with lipidated LC3, which is the LC3 that sits on autophagosomes, by binding to its lipid moiety. There are a large number of autophagy regulators and we've added one more into the mix. The question of why this gene is taxonomically restricted and what roles it plays in tissues like liver and brain need answers. In general, it is more difficult to study lipids than proteins. The work carried out in this paper doesn't really fall within the primary area of research in our lab, which is yeast biology, but I believe we've done our best to tease apart the basic properties of this protein. Someone, somewhere, should knock out this protein in mice and see what happens.

**What has surprised you the most while conducting your research?**

In my project, the very first breakthrough was a pleasantly surprising one. Researchers all over the world use liposome-based transfection for protein expression; we do it too. However, ApoL9 reacts with a lipid in transfection reagents and aggregates. So, we were initially working under the impression that the protein was inherently aggregation-prone. It took us quite a lot of head-scratching and double takes to realise what was happening, and that what we were seeing under the microscope was an artefact. That was the end of the lipid transfections, but the start of my project! Another thing that's surprised me is how the human mind can sometimes be tempted by biases and assumptions to construct very believable, seemingly solid hypotheses. These hypotheses often fall apart dramatically, leaving one wondering how we even thought of them in the first place! With time, surprise is slowly replaced by caution and a sense of battle-weariness.

**What, in your opinion, are some of the greatest achievements in your field and how has this influenced your research?**

I haven't really worked in a particular field yet; however, CRISPR gene editing is definitely a huge leap, though I haven't had the chance to use it. That's why I feel all those people working with non-conventional model organisms on topics that superficially seem obscure might be doing very important work; one never knows what will be discovered where. There has also been tremendous progress in various microscopy techniques, but I feel the technologies sometimes take time to become widely available and affordable, especially in developing countries where funding for science is meagre.

**Figure:**
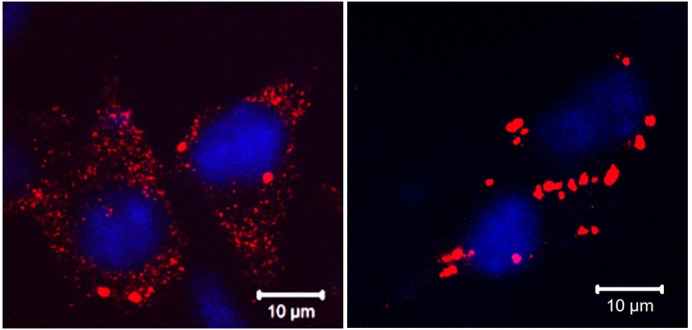
**B16F10 mouse melanoma cells stably expressing V5-tagged Apolipoprotein L9.** Cells without treatment (left) and cells transfected with an empty plasmid using a liposome-based reagent (right). ApoL9 interacts with the helper lipid DOPE in the transfection reagent, triggering aggregation.

“I feel all those people working with non-conventional model organisms on topics that superficially seem obscure might be doing very important work; one never knows what will be discovered where.”

**What changes do you think could improve the professional lives of early-career scientists?**

To start with, people should first have the chance to become an early-career scientist! I speak for people from countries like mine; in this case, India. There are a load of PhDs graduating every year, with very few opportunities available for postdoctoral research. This is improving ever so slightly, but ultimately government funding for science holds all the keys. As of now, PhDs here are, by default, forced to look abroad for better research opportunities. This brings with it its own set of complications and challenges. This must change. But I'm not very optimistic. Better pay and permanence of jobs can come later. First, the infrastructure must be created.

**What's next for you?**

Currently, I'm tidying up things after completing my PhD work, taking a second look at a couple of interesting problems I didn't have the time to look into before. I really want to continue doing research. Because I've not really worked on a narrow research area so far, I hope I can choose an interesting field to continue in. For that, I plan to do some reading. Foresight is probably the most useful skill a person can have, and it's a skill that I, so far, have completely lacked! I'm interested in writing and journalism, but I have no qualifications whatsoever in those directions. Industry jobs don't appeal to me. Please reach out to me if you feel I could fit in your laboratory/workplace!

**What attitudes could change within the scientific community, regarding how science is done and viewed?**

I believe people must be evaluated and employed on the basis of the work they do, not which journals they publish in. Sometimes people ask me what the impact factor of this or that journal is, and I say I don't know. That's because I usually don't really know. I don't think there is much point in remembering these numbers, is there? Very often, I see people rattling off the names of all the journals that a researcher has published in, but they wouldn't have bothered to open those papers and look at what the research was about! This attitude must change, because it spoils the system from the bottom up. The moment one starts to give more importance to the status/name of a journal than the science contained in it, they're losing the plot. Also, people could be nicer to each other. It speeds up progress.
